# Spatially gridded cross-shelf hydrographic sections and monthly climatologies from shipboard survey data collected along the Newport Hydrographic Line, 1997–2021

**DOI:** 10.1016/j.dib.2022.107922

**Published:** 2022-02-04

**Authors:** Craig M. Risien, Melanie R. Fewings, Jennifer L. Fisher, Jay O. Peterson, Cheryl A. Morgan

**Affiliations:** a College of Earth, Ocean, and Atmospheric Sciences, Oregon State University, Corvallis, OR, 97331, United States of America; b Cooperative Institute for Marine Ecosystem and Resources Studies, Oregon State University, Newport, OR, 97365, United States of America; c NOAA Office of Science and Technology, Silver Spring, Maryland, 20910, United States of America

**Keywords:** Seawater temperature, Practical salinity, Potential density, Spiciness, Dissolved oxygen, Climatology, California current, Oregon

## Abstract

The Oregon continental shelf is embedded within the northern California Current System, a wind-driven, eastern boundary system that includes the equatorward flowing California Current and the poleward flowing California Undercurrent. During spring and summer months, equatorward winds drive the upwelling of cold, nutrient-rich, and oxygen-poor waters from depth onto the shelf, fueling a highly productive marine ecosystem that supports several valuable commercial fisheries. This data article describes a time series of hydrographic data collected on a biweekly to monthly schedule from March 1997 to July 2021 along the Newport Hydrographic Line (NHL; 44.652°N, 124.1 – 124.65°W) located west of Newport, Oregon. The NHL, with its 2–4 week sampling rate and inclusion of biological data such as zooplankton net tows, is the only long-term, high-frequency dataset of its kind for the California Current and as such is crucial to understanding the connectivity between changes in ocean-climate and ecosystem structure and function. Data were collected using Sea-Bird Scientific conductivity, temperature, depth (CTD) profilers with associated dissolved oxygen sensors at seven stations located between 1.9 and 46.3 km from shore. Water depths for the seven stations range from 30 to 296 m. Data collected during each cruise were processed using Sea-Bird Scientific's Seasoft software package. These CTD station data were gridded to a 0.01° x 1 dbar longitude - pressure grid using linear interpolation to create cross-shelf hydrographic sections of temperature, practical salinity, potential density, spiciness, and dissolved oxygen. From the gridded section data, seasonal climatologies were calculated for each variable at each location in the longitude - pressure section using harmonic analysis with a three-harmonic fit to the gridded transect observations. The station data, gridded transect data and monthly climatologies for all five variables are available via Zenodo at https://doi.org/10.5281/zenodo.5814071.

## Specifications Table

**Table:** 

Subject	Oceanography
Specific subject area	Hydrographic data collected off Newport, Oregon (44.65°N, 124.1 – 124.65°W)
Type of data	Hydrographic Figure
How data were acquired	CTD data were collected on a biweekly to monthly schedule from March 1997 to July 2021 along the Newport Hydrographic Line. Dissolved oxygen observations were routinely collected between August 1998 and July 2021. These data were acquired using Sea-Bird Scientific 19 SeaCAT, 19plus V2 SeaCAT, SBE 25 Sealogger, or SBE 25plus Sealogger CTD (conductivity, temperature, depth) profilers with associated SBE 43 dissolved oxygen sensors. They were processed using Sea-Bird Scientific's Seasoft software package.
Data format	Raw Analyzed
Description of data collection	This data set consists of CSV files that contain hydrographic station data and NetCDF files that contain interpolated, cross-shelf hydrographic sections for each cruise; derived climatologies, calculated using harmonic analysis; and associated linear regression coefficients. It also includes example MATLAB® and R scripts that show how to read the data files, plot cross-shelf sections, and calculate climatologies using the provided regression coefficients.
Data source location	Hydrographic data collected off Newport, Oregon Station Latitude Longitude Water Depth (m) NH-01 44.652°N 124.100°W 30 NH-03 44.652°N 124.130°W 48 NH-05 44.652°N 124.177°W 60 NH-10 44.652°N 124.295°W 81 NH-15 44.652°N 124.412°W 90 NH-20 44.652°N 124.528°W 140 NH-25 44.652°N 124.650°W 296
Data accessibility	Repository name: Zenodo Data identification number: 10.5281/zenodo.5814071 Direct dataset link: https://doi.org/10.5281/zenodo.5814071

## Value of the Data

• The Newport Hydrographic Line (NHL) is the only regular, long-term (multiple decades), high-frequency (more frequent than quarterly) ship-based CTD section dataset sampling across the full continental shelf for the California Current System and as such is a crucial resource for understanding the connectivity between changes in ocean-climate and ecosystem structure and function [1,2].
• The gridded, quality-controlled NHL observations presented here provide valuable information to support resource managers and marine scientists facing critical and emerging issues that are expected to be exacerbated by climate change, including marine heatwaves, ocean acidification, hypoxia, and harmful algal blooms [3], [4], [5], [6].
• With more than 24 years of biweekly to monthly observations, the data presented here can, for example, be used to better understand ecosystem change and the influence of basin-scale forcing on coastal upwelling at intra-seasonal, seasonal, interannual, and decadal time scales.

## 1 Data Description

**Fig. 1:**
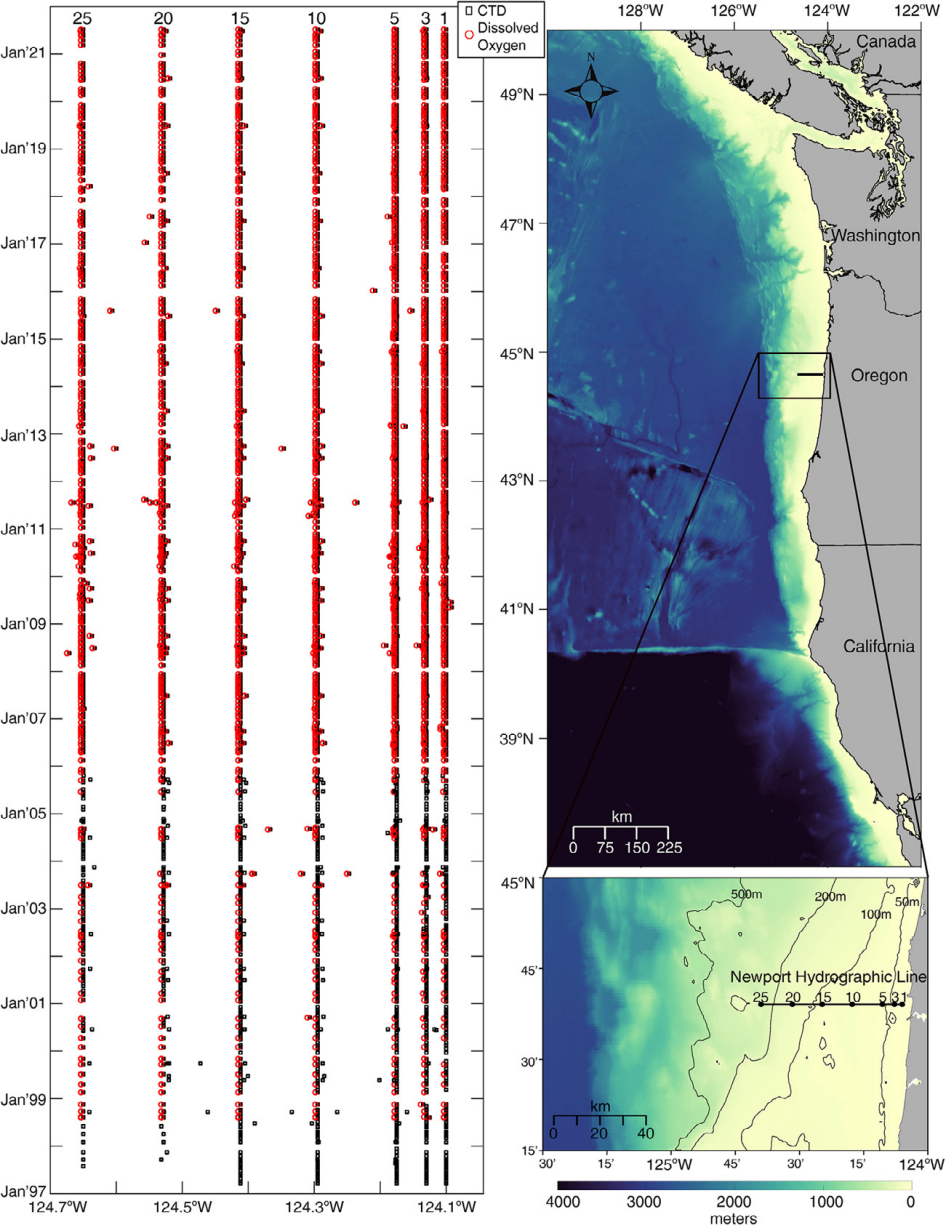
The left panel shows when CTD (black squares) and dissolved oxygen (red circles) measurements were made at stations NH01, NH03, NH05, NH10, NH15, NH20, and NH25 between March 1997 and July 2021. The top right panel shows GEBCO bathymetry [7] off the West Coast of North America and the Newport Hydrographic Line (NHL) located west of Newport, Oregon. The lower right panel shows the NHL stations. Station numbers correspond to the station distance from shore, in nautical miles. The 50, 100, 200, and 500 meter isobaths are shown.

Newport Hydrographic Line station data; gridded, cross-shelf hydrographic sections; and derived monthly climatologies for temperature, practical salinity, potential density, spiciness, and dissolved oxygen described here are available via Zenodo at https://doi.org/10.5281/zenodo.5814071. The data set consists of CSV (Comma Separated Values) files (*newport_hydrographic_line_station_data.zip*) that contain CTD and dissolved oxygen observations collected at the seven hydrographic stations located 1, 3, 5, 10, 15, 20 and 25 nautical miles west of Newport, Oregon between March 1997 and July 2021 (Fig. 1). Additionally, the data set contains three NetCDF files that follow CF (Climate and Forecast) metadata conventions: *newport_hydrographic_line_gridded_sections.nc* contains observations gridded to a 0.01° × 1 dbar longitude - pressure grid to create cross-shelf hydrographic sections for each of the five variables for each cruise. *newport_hydrographic_line_gridded_section_climatologies.nc* contains climatological hydrographic sections, calculated using harmonic analysis over the 24-year period March 1997 to February 2021 for temperature, practical salinity, potential density, spiciness, and the 22.6-year period August 1998 to February 2021 for dissolved oxygen. Monthly climatology values are reported here as an average of the daily climatology values for each month. *newport_hydrographic_line_gridded_section_coefficients.nc* contains the associated three-harmonic linear regression model coefficients for all five variables. From the regression coefficients, users can construct seasonal cycles at any location in the gridded section with a temporal resolution that best suits their specific needs.

## 2 Experimental Design, Materials and Methods

### 2.1 Data collection and interpolation

For each of the 556 NHL research cruises completed between March 1997 and July 2021, data were collected using Sea-Bird Scientific 19 SeaCAT, 19plus V2 SeaCAT, SBE 25 Sealogger, or SBE 25plus Sealogger CTD (conductivity, temperature, depth) profilers with associated SBE 43 dissolved oxygen sensors at hydrographic stations located between 1.9 and 46 km west of Newport, Oregon. Data collected during each cruise were processed using Sea-Bird Scientific's Seasoft software package and binned to 1 dbar pressure or 1 m depth bins. Bin depths were converted to pressure (dbar) using the Gibbs-SeaWater Oceanographic Toolbox [8]. Binned temperature, practical salinity and pressure station data were used to calculate potential density and spiciness at each station using the Gibbs-SeaWater Oceanographic Toolbox.

**Fig. 2:**
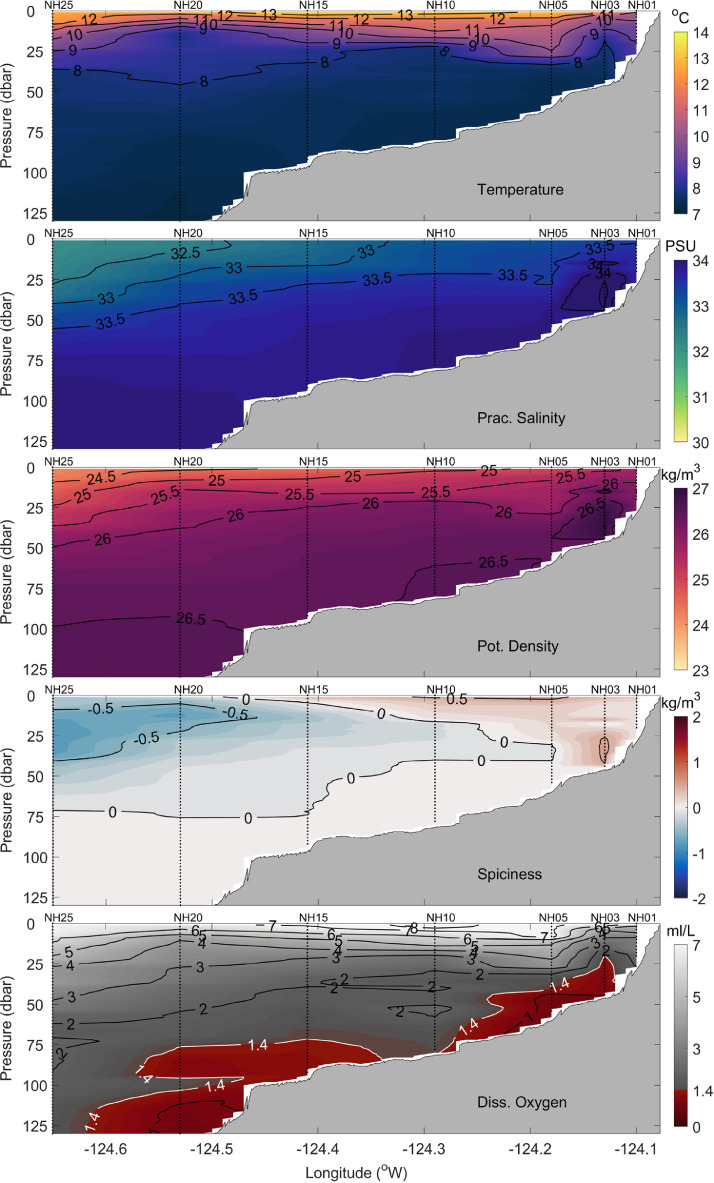
Example of spatially interpolated cross-shelf hydrographic sections of temperature, practical salinity, potential density, spiciness, and dissolved oxygen (top to bottom panels, respectively) collected along the Newport Hydrographic Line on 8 July 2021. Stations NH01, NH03, NH05, NH10, NH15, NH20, and NH25 where CTD casts were conducted are shown as vertical dotted lines.

**Fig. 3:**
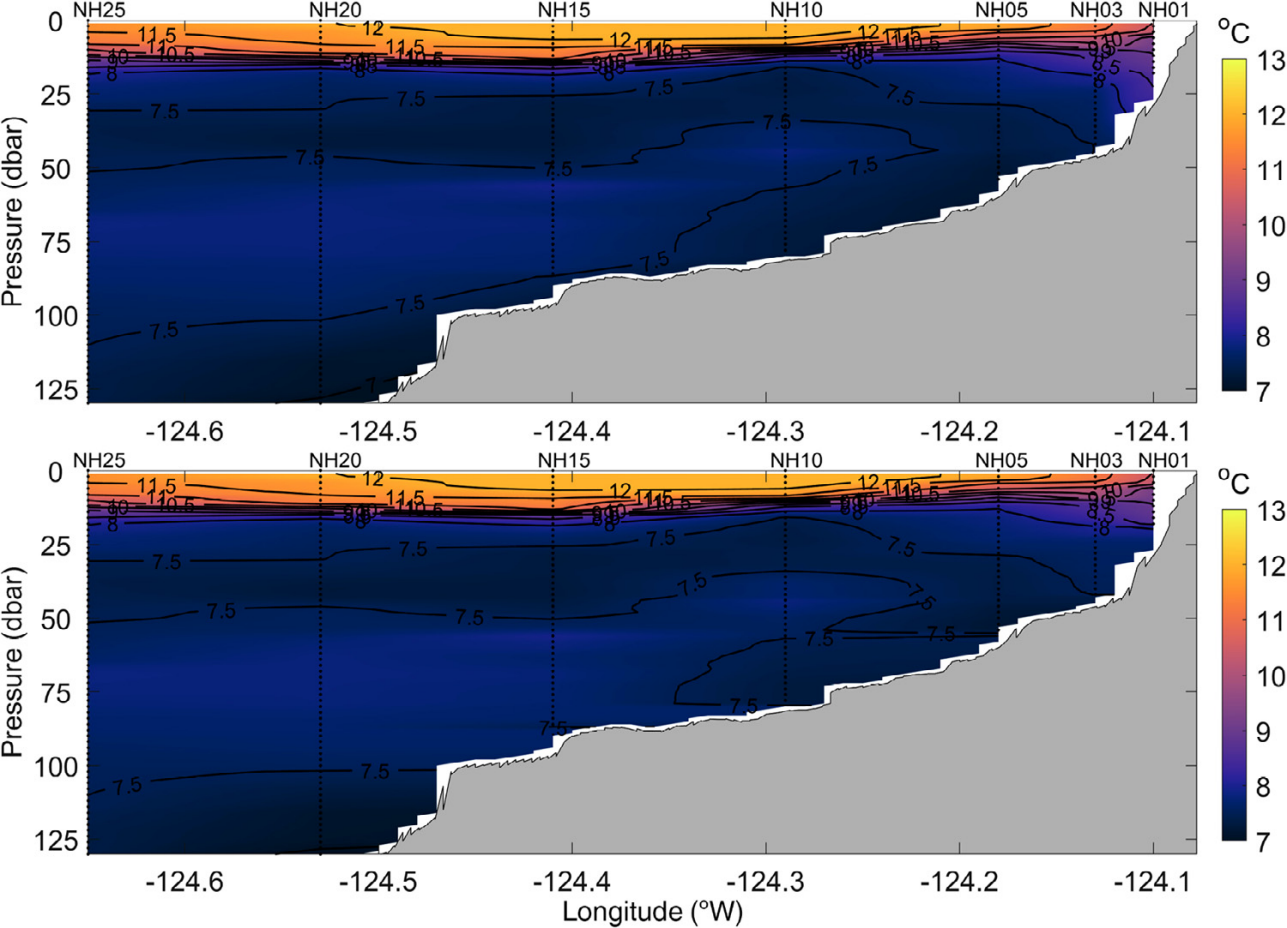
Spatially interpolated cross-shelf hydrographic sections of temperature data collected on 12 June 2008. The top panel shows interpolation results based on the MATLAB ‘scatteredInterpolant’ function using the linear method. The lower panel shows interpolation results based on the ordinary kriging (using a linear variogram model) method. Stations NH01, NH03, NH05, NH10, NH15, NH20, and NH25 where CTD casts were conducted are shown as vertical dotted lines.

**Fig. 4:**
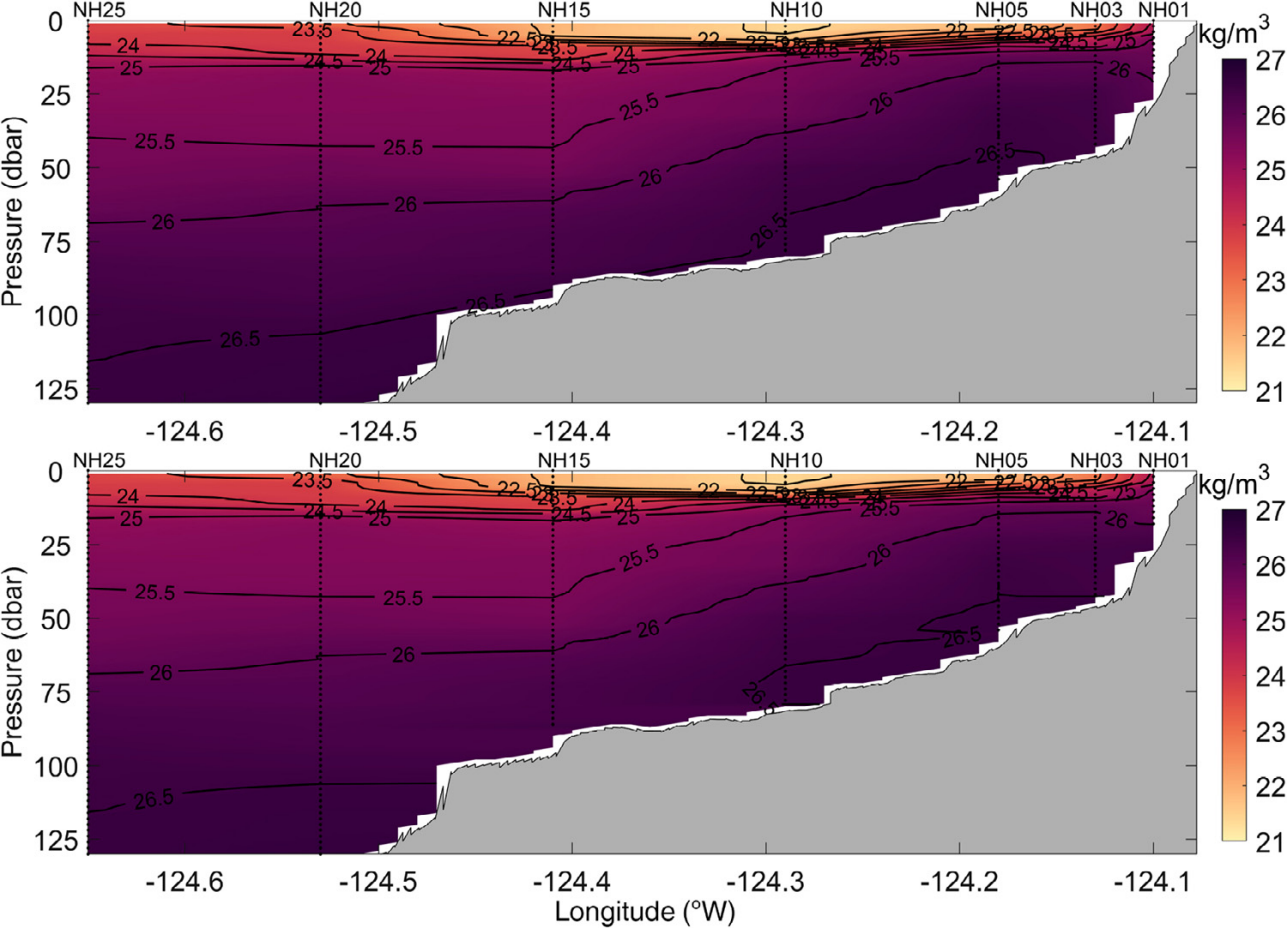
Same as Fig. 3, but for potential density.

**Fig. 5:**
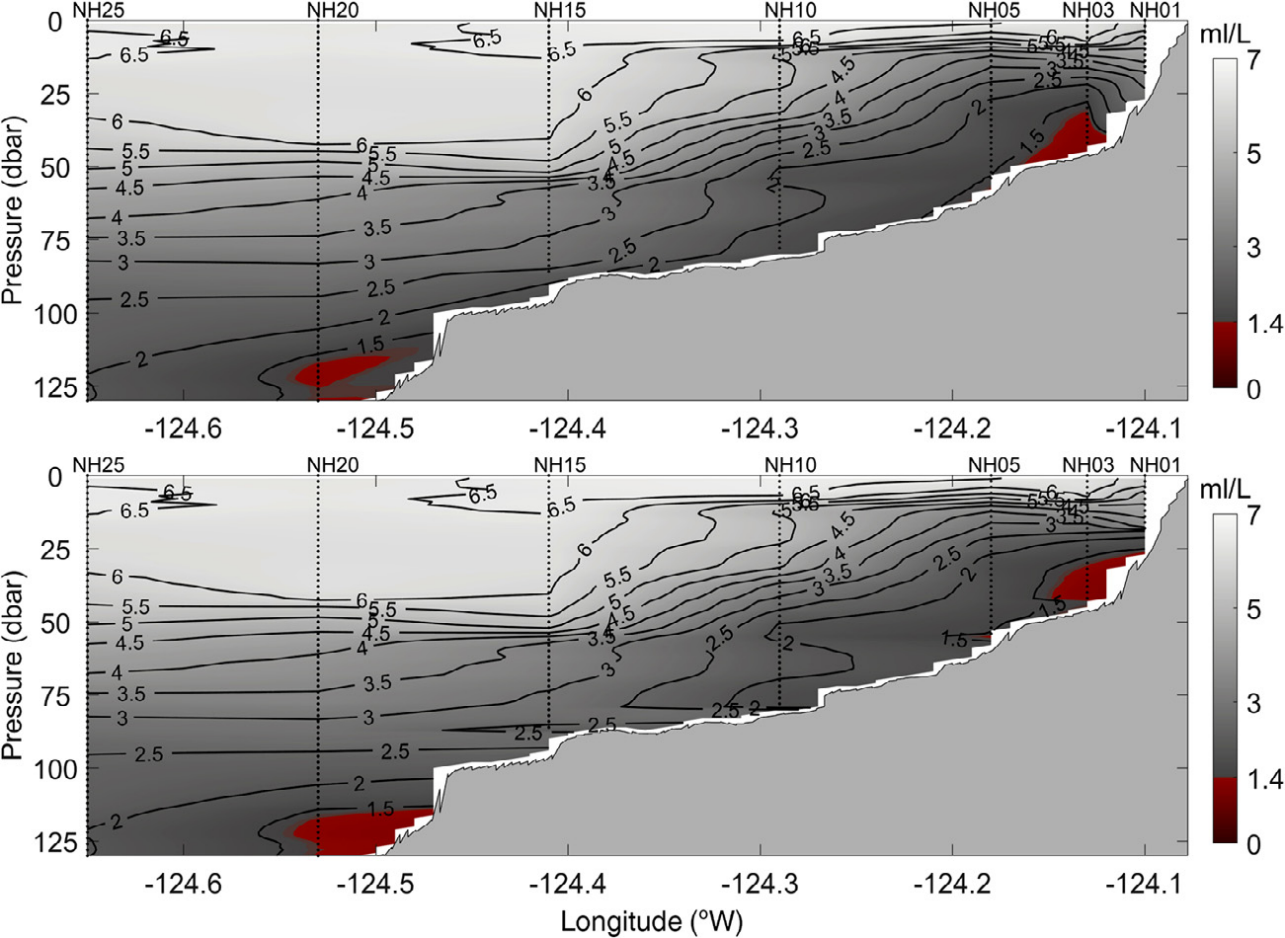
Same as Fig. 3, but for dissolved oxygen.

All station data were interpolated to a 0.01° × 1 dbar longitude - pressure grid to form complete cross-shelf sections of temperature, practical salinity, potential density, spiciness, and dissolved oxygen. Fig. 2 shows an example of gridded cross-shelf sections of temperature, practical salinity, potential density, spiciness, and dissolved oxygen for data that were collected on 8 July 2021. Data were gridded using the MATLAB® ‘scatteredInterpolant’ function with the *linear* interpolation method. The sensitivity of the results to using other interpolation methods, including the ‘scatteredInterpolant’ function with the *natural neighbor* or *nearest neighbor* method, was evaluated. The *natural neighbor* method generated very similar results to the *linear* method (not shown). The *nearest neighbor* method produced blocky, unrealistic looking cross-sections that contained numerous discontinuities (not shown). Ordinary kriging interpolation [9] was investigated using a variety of variogram models. The results of the kriging using a linear variogram model are shown in Figs. 3, 4, and 5 for temperature, potential density, and dissolved oxygen, respectively. While the more complex and computationally expensive kriging method with a linear variogram model generated similar results to linear interpolation in the upper water column, it produced unrealistic results near the bottom, tending to create near-horizontal contour lines (e.g., in Fig. 3 bottom panel, the 7.5 °C contour between stations NH10 and NH05; and in Figs. 4 and 5 bottom panels, values near the bottom between stations NH10, NH15, and NH20). Finally, a thin-plate spline method using the MATLAB® function ‘tpaps’ with various smoothing parameterizations was considered, but this method produced unrealistically smooth results. While our analysis shows the MATLAB® ‘scatteredInterpolant’ function using the *linear* method to be well suited for the interpolation of the cross-shelf hydrographic sections, there are many different approaches to data interpolation and the optimal choice may vary depending on the application. The original binned CTD station data are therefore included in the NHL Zenodo data set to allow users to create gridded cross-shelf sections using their preferred interpolation method should they desire.

### 2.2 Transect climatologies

Each cross-shelf hydrographic section, collected over a period of several hours, was mapped to a particular year-day based on the start date of the associated cruise to create a “daily” data set. The daily data set was then used to calculate climatologies, based on the 24-year period March 1997 to February 2021 for temperature, practical salinity, potential density, and spiciness, and based on the 22.6-year period August 1998 to February 2021 for dissolved oxygen, using harmonic analysis at each location in the longitude - pressure section. An advantage of harmonic analysis over other methods for calculating climatologies, such as long-term monthly averages, is that the resulting regression coefficients allow for the calculation of climatological values at any arbitrary time of month or year.

**Fig. 6:**
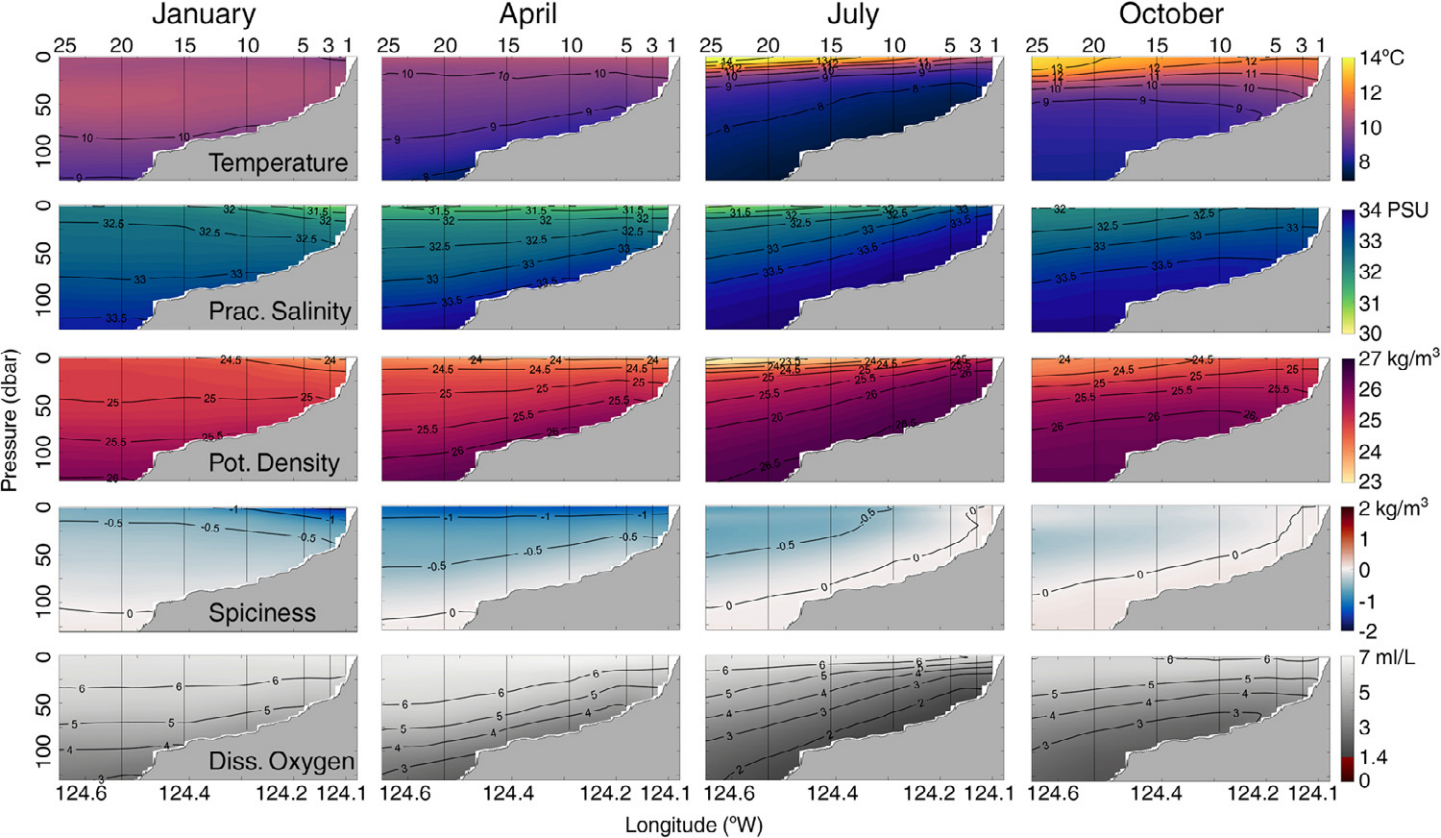
January, April, July and October temperature, practical salinity, potential density, spiciness and dissolved oxygen climatology transects along the Newport Hydrographic Line. The temperature, practical salinity, potential density, spiciness climatologies are based on the 24-year period March 1997 to February 2021. The dissolved oxygen climatology is based on the 22.6-year period August 1998 to February 2021. The values shown here are monthly averages of the climatology.

The climatologies presented here (Fig. 6) are based on a three-harmonic fit (1, 2, and 3 cycles per year) to the daily, gridded interpolated transect observations. The multiyear time series at each longitude - pressure grid location in the cross-shelf section was fitted to a seven-parameter regression model consisting of a constant plus three harmonics. This linear regression model can be written as follows, here for the temperature climatology *T_clim_*:

$$\begin{array}{ccc} T_{clim}\left(x,z,t\right) & = & {\hat{\beta }}_{0}+{\hat{\beta }}_{1}sin\left(2\pi ft\right)\;+{\hat{\beta }}_{2}cos\left(2\pi ft\right)\;+{\hat{\beta }}_{3}sin\left(4\pi ft\right)\;+{\hat{\beta }}_{4}cos\left(4\pi ft\right)\\ & & +\;{\hat{\beta }}_{5}sin\left(6\pi ft\right)+{\hat{\beta }}_{6}cos\left(6\pi ft\right) \end{array}$$

where *x* is cross-shelf location, *z* is vertical location, *t* is time, the ${\hat{\beta }}_{m}$ are the best-fit parameters determined by linear regression to the observed time series at that location and time *T(x,z,t)*, and *f* = 1/(365.2422 dy), one cycle per year. The decision to use a three-harmonic fit was based on an assessment of the extra sum of squares test results for significance of the higher harmonics, and whether the regression coefficient values were significantly different from zero with 95% confidence.

## Ethics Statement

The data sets described here involved no human subjects, animal experiments or social media platforms.

## CRediT Author Statement

**Craig Risien:** Writing – Original draft preparation, Data processing and Analysis; **Melanie Fewings:** Supervision, Analysis, Writing– review & editing; **Jennifer Fisher:** Data collection and processing, Writing – review & editing; **Jay Peterson:** Data collection and processing, Writing – review & editing; **Cheryl Morgan:** Data collection and processing, Writing – review & editing.

## Declaration of Competing Interest

The authors declare that they have no known competing financial interests or personal relationships which have or could be perceived to have influenced the work reported in this article.
